# Epidemiology of Football-Related Sudden Cardiac Death in Turkey

**DOI:** 10.3390/medicina57101105

**Published:** 2021-10-14

**Authors:** Ali Işın, Adnan Turgut, Amy E. Peden

**Affiliations:** 1 Department of Coaching Education, Faculty of Sports Sciences, Akdeniz University, Antalya 07070, Turkey; aliisin@akdeniz.edu.tr; 2 Department of Physical Education and Sports, Faculty of Sports Sciences, Akdeniz University, Antalya 07070, Turkey; turgut@akdeniz.edu.tr; 3 School of Population Health, Faculty of Medicine, University of New South Wales, Sydney, NSW 2052, Australia

**Keywords:** football, sport, prevention, injury, mortality

## Abstract

*Background and Objectives*: Sudden cardiac death (SCD), particular among elite footballers, has attracted much attention in recent times. However, limited information exists on football-related SCD in Turkey. Autopsy-based studies of sports-related sudden deaths in Turkey are rare and often have small sample sizes. To address this, this study aimed to determine the population-based incidence and profile of football-related SCD nationally in Turkey. *Materials and Methods*: Due to a lack of national data on this issue, football-related SCD (non-elite competitive or recreational football) between 1 January 2011, and 31 December 2019 were identified by dual, independent identification and screening of online media reports. Deaths were explored by sex, age group, season, and phase of exercise. Descriptive statistics were utilised. Age-specific mortality rates and proportional mortality rates were calculated. *Results:* In total, 118 football-related SCD were identified, a crude mortality rate of 0.41 per 100,000 population. All fatalities were males and the mean age was 35.5 years ± 10.4. Those aged 40–49 years recorded the highest mortality rate (0.67/100,000), three times the risk of those aged 50–59 years (RR = 3.1; 95%CI:1.5–6.4). Those aged 30–39 recorded the highest age-specific proportional mortality rate (0.86/1000 deaths). The highest risk occurred while playing football (*n* = 97; 82.2%), with another 15% of deaths (*n* = 18) occurring within 1 h of play. Almost all fatalities (*n* = 113; 95.8%) occurred during participation in recreational football. *Conclusions:* This study has identified football-related SCD most commonly occurs during recreational football among males aged 30–49 years. It is recommended males of this age participating in recreational football be encouraged to seek pre-participation heart health checks. Given the value of automated external defibrillators (AEDs) in responding to out-of-hospital cardiac arrest, future research should explore the feasibility and effectiveness of AEDs in preventing football-related SCD in Turkey including training of first responders in cardiopulmonary resuscitation and AED use.

## 1. Introduction

Football (also known as soccer) is the most popular sport in the world with an estimated 260 million participants [1]. As in all sports however, there are risks, such as the risk of sudden death. Sudden deaths of top footballers such as Miklós Fehér, Marc-Vivien Foe, Daniel Jarque and Antonio Puerta have all attracted attention in recent years [2]. Such deaths are all the more remarkable because football players are considered extremely fit and healthy [3]. More recently, the high-profile cardiac arrest and successful resuscitation of Danish footballer Christian Eriksen during the Euro 2020 championships have again attracted global attention to this issue [4].

Sudden cardiac death, although uncommon, can occur during sports and physical activity and includes unexpected deaths in people with symptoms onset within one hour of their death [5,6]. Some risk factors such as age, gender and type of activity have been found in sudden deaths related to sports, including increased prevalence among males, likely due to increased participation and differences in training frequency and intensity [7,8]. 

More than half of all sudden deaths in athletes are due to cardiovascular causes, the most common of which are hypertrophic cardiomyopathy and congenital coronary artery anomalies [6]. A study published in 2017 identified hypertrophic cardiomyopathy as most common among athletes under 35 years of age [9], although it must be noted this pathology is becoming increasingly rare as a cause of SCD in athletes due to pre-participation cardiac evaluation and athlete exclusion from continuing competition. Coronary artery disease is the most common cause of sudden death in athletes aged 35 years and older [10,11,12]. 

Football is a sport commonly implicated in sudden deaths. Almost half (44.9%) of all sudden cardiac deaths in a study from Italy occurred during football [13], while 39% of sport-related sudden cardiac deaths in Israel involved football [14]. Most sports-related deaths in Germany were cardiac-related, with football and track and field commonly implicated sports [15]. Suerez et al. [16] reported that 21.3% of sudden sport-related deaths in Spain between 1995 and 2001 occurred while playing football. 

Despite football being a popular sport in Turkey, there is scarce participation data and very little information on the incidence of sudden death among footballers [17]. Autopsy-based studies of sports-related sudden deaths in Turkey are rare and often have small sample sizes [17,18]. To address this gap in the literature, this study aimed to investigate the population-level incidence and profile of football-related sudden cardiac death nationally in Turkey between 2011 and 2019.

## 2. Materials and Methods

As autopsies are not performed for every death in Turkey [17], relying on official data will underreport the total number of cases [18]. Due to a lack of official data at a national level, all data presented in this study were identified from media reports. This is an accepted approach and has been used previously to determine the incidence of sudden cardiac death in athletes [10,19,20,21]. However, it must be noted that inclusion of cases within this study rely on the media reporting cardiac involvement, as opposed to a sudden (unexplained) death of other causes. 

### 2.1. Study Design

This study investigating football-related sudden cardiac deaths in Turkey using a retrospective and web-based design. Media reports were used to identify and determine the incidence of football-related sudden cardiac deaths in this study. This study was approved by The University of New South Wales Human Research Ethics Committee on the 29 September 2021 (HC210766) and conducted in accordance with Helsinki Declaration.

### 2.2. Study Population

The study involved deaths which occurred in Turkey during the study period and were reported in the media as being related to recreational or non-elite competitive football and were triggered by cardiac causes No lower or upper age limit was set for the study sample population. 

### 2.3. Data Collection

To identify cases of football-related sudden cardiac death which occurred between 1 January 2011 and 31 December 2019 retrospective screening was performed of online media reports via the search engine Google News (www.news.google.com). The following search terms in Turkish language were used: *“sudden death” (“ani ölüm”)* OR *“sudden cardiac death” (“ani kardiyak ölüm”)* OR *“cardiac death” (“kardiyak ölüm”)* OR *“heart attack” (“kalp krizi”)* OR *“cardiac arrest” (“kalp durması/kardiyak arrest”* OR *“death” (“ölüm”)* AND *“football” (“futbol”)* OR *“football pitch” (“futbol sahası”)* OR *“astroturf pitch”. (“halı saha”)* In addition, we strengthened our search results by using the following phrases in Turkish language in a general Google search (www.google.com) to identify media reports not found via Google News searches: *“sudden death in football”, (“futbolda ani ölüm”) “sudden death in football field”, (“futbol sahasında ani ölüm”) “cardiac death in football”(“futbolda kadiyak ölüm”), “cardiac death in football field” (“futbol sahasında kardiyak ölüm”), “heart attack in football” (“futbolda kalp krizi”), “heart attack in football match”, (“futbol maçında kalp krizi”) “cardiac arrest in football” (“futbolda kalp durması/kardiyak arrest”), “cardiac arrest in football match” (“futbol maçında kalp durması/kardiyak arrest”), and “death in football match” (“futbol maçında ölüm”).*

Searches were run and the data were collected and recorded independently by two researchers (A.I. and A.T.). The researchers then compared data to ensure completeness of data and accuracy of coding. Duplicate cases were removed. Cases with missing data, or an unidentified cause of death, were not included in the study. Because media reports were updated periodically, all data were last reviewed on 22 June 2020.

As previously reported by Bohm et al. [10], we defined football-related sudden cardiac death as a cardiac-related death that occurred during or within 1 h of football participation (before or after). The timing of sudden cardiac death was evaluated in three groups by phase of exercise: before exercise (warm-up phase), during exercise (the phase in which football is played), after exercise (the first hour after the end of exercise). The level of football participation was divided into two categories, non-elite competitive and recreational football. Date of death was used to classify season of incident. All data were presented by division into the following five age groups: 10–19 years, 20–29 years, 30–39 years, 40–49 years, and 50–59 years. No deaths were recorded in those aged under 10 years or 60 years or older. 

### 2.4. Data Analysis

All data were transferred from online Google News searches into Microsoft Excel 365 ^TM^ (Redmond, Washington, USA) and then into IBM SPSS V23 (Armonk, NY, USA for statistical analysis. For all variables, analysis comprised calculation of the frequency and percentage in each category (i.e., age group). For age group, the mean and standard deviation was calculated. Data on the resident population of Turkey (males aged 10–59 years) by age group were sourced from the Turkish Statistical Institute [22] and used to calculate age-specific mortality rates as per the following formula as specified by Koepsell and Weiss (2014) [23]. 

Age-cause-specific mortality rate:

$$\frac{n\;of\;football-related\;SCDs\;\;among\;individuals\;in\;a\;specific\;age}{population\;of\;specific\;age}\times 100,000$$

Data on all-cause mortality (for males aged 10–59 years) by age group were also obtained from the Turkish Statistical Institute [22] and used to calculate mortality rates using the following formula as specified by Koepsell and Weiss (2014) [23].

$$\mathrm{The}\;\mathrm{proportional}\;\mathrm{mortality}:\;\frac{n\;of\;deaths\;from\;football-relatedSCD}{n\;of\;deaths\;from\;all\;causes}\times 1000$$

The relative risk (RR) and 95% confidence intervals (95% CI) were calculated using population data for the age groups. The age group with the lowest number of cases (50–59 years) was considered as the reference point for calculating the RR and 95% CI. 

## 3. Results

**Figure 1:**
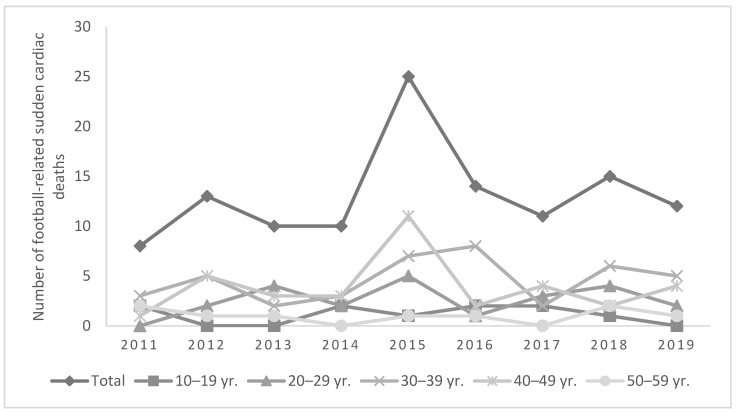
Number of football-related sudden cardiac death in Turkey by year (2011–2019). Yr: years of age.

From 2011 to 2019, 118 football-related sudden cardiac deaths in Turkey were identified from media reports. The average number of football-related sudden cardiac death per year during this 9-year period was 13 (range 8–25) (Figure 1). This equates to an average annual mortality rate of 0.41 per 100,000 people. All football-related sudden cardiac deaths were males (100.0%) and aged between 13 and 59 years (mean age 35.5 SD ± 10.4). 

One third (34.7%; *n* = 41) of football-related sudden cardiac deaths occurred in individuals aged 30–39 years, followed by 40–49 year-olds (29.7%; *n*: 35). The highest age-specific mortality rate occurred among 40–49year-olds (0.67 per 100,000 population) and the lowest rate was in the age group 10–19 years (0.15 per 100,000). When considering all-cause mortality, the football-related sudden cardiac death rate was 0.30 per 1000 deaths (all causes of death). This rate was found to be lowest in the 50–59 age group (0.05 per 1000 deaths). The relative risk and confidence interval for football-related sudden cardiac death varied depending on the age of the individuals participating in football. The age group 40–49 years had the highest risk (RR = 3.1; 95%CI: 1.5–6.4) compared to the reference age group 50–59 years (Table 1).

The vast majority of football-related sudden cardiac deaths occurred during exercise, accounting for 82.2% of all cases (*n* = 97), followed by after exercise (15.3%; *n* = 18). Just 2.5% (*n* = 3) occurred before exercise. Among 10–19 year-olds, no cases were found before or after exercise. Similarly, in the age group 40–49 years, no cases were noted before exercise. While 113 cases (95.8% of all cases) occurred during recreational football, five cases (4.2%) were associated with non-elite competitive football (Table 2).

Percentage and incidence of football-related sudden cardiac deaths in Turkey by season is given in Table 3. Spring (29.7%) followed by Autumn (24.6%) are the seasons with the highest proportions of football-related sudden cardiac deaths. When considering age group, the largest proportion of deaths among 20–29 year-olds occurred in Spring (*n* = 8; 34.8%), whereas Summer saw the largest proportion of deaths among 50–59 year-olds (*n* = 4; 44.4%) (Table 3).

## 4. Discussion

Although participation in football is a popular past time with many health and social benefits [24,25], it is not without risk of harm such as sudden death. High-profile sudden deaths of footballers, who were considered to be particularly healthy [3] and the limited information on the topic in Turkey [17] prompted researchers to investigate football-related sudden cardiac death in Turkey.

In the present study, football-related age-specific mortality was found to be 0.41 per 100,000 population and proportional mortality was 0.30 per 1000 deaths. This compares favorably with previously published studies identifying mortality rates of 0.9 in physically active men aged 15–34 years in Norway [26], but is higher than studies of sudden cardiac death among athletes in Italy (0.28) [21]. When compared to published rates of football-related sudden cardiac death, our finding (0.41) is lower [3,27]. The incidence of sudden cardiac death in athletes indicates controversial results, largely due to the fact that studies attempt to determine the incidence of sudden cardiac death using different calculations [28]. Football-related sudden cardiac death, though rare, is a problem that requires attention [21]. 

In Turkey, health examinations are mandatory for professional players, but there are no regulations for non-professional football players and recreational football players. Furthermore, professional football players undergo more in-depth health examinations compared to non-professional football players. A medical examination is required for amateur football players only when a football license is first issued [29]. The degree of medical examination of amateur license holders is in sharp contrast to that of professional footballers. According to the Turkish Football Federation, every professional footballer is required to pass the blood pressure, 12-lead ECG, cardiac stress test and echocardiogram before the season. Thus, possible heart problems in professional football players are detected in advance and the necessary measures are taken [30].

In an autopsy-based study of football-related sudden cardiac deaths in Turkey between 2000 and 2005, 15 cases aged 10–48 years were reported. Thirteen of these cases occurred during recreational football [18]. Similarly, in the study investigating deaths in sports and recreational activities in Turkey, six cases were reported, five of which were related to football, the youngest being 18 years old and the oldest 43 years old [17]. Most of the reported football-related cases were cardiac in origin, and all deaths were caused by coronary artery disease [17]. These two reported studies had relatively small sample sizes due to the use of data derived from autopsies, which are not routinely conducted in Turkey [17,18]. Therefore, the incidence of sudden cardiac death associated with football could not be calculated. Although a comparison with our current results regarding the incidence of football-related cardiac death is not possible, it can be concluded that almost all football-related sudden cardiac deaths in Turkey occur in recreational football, a finding confirmed in the present study. Consideration should be given to the feasibility of screening individuals participating in recreational football to determine cardiac health [17]. In this way, possible cardiac problems can be detected in advance and the necessary measures can be taken. This has been conducted among competitive athletes in Italy to great effect, reducing mortality among young competitive athletes by 90%, mostly by the prevention of sudden death from cardiomyopathy [8,31,32].

Our research found that nearly two-thirds of football-related deaths in Turkey occurred in males aged 30 to 49 years. This is a similar finding to the two previously published autopsy-based studies in Turkey which found people aged 30 years and older accounted for 47% and 80% of deaths respectively [17,18]. These findings are also similar to those reported in other countries. Most sports-related deaths in Germany occur in middle-aged men [10] and more than half of the sports-related sudden cardiac deaths in Spain occurred while playing football among people between the ages of 30 and 54 years [33]. There is no evidence to support any medical investigation being useful in this age group. There may, however, be value in encouraging healthier lifestyles including diet and exercise and a reduction in the use of illicit substances and tobacco [34,35]. 

Our finding that football-related sudden cardiac death in Turkey occurs most commonly during exercise (82%) is similar to that of other previously published studies [10,36]. As these results suggest, exercise may lead to an increased risk of sudden cardiac death or cardiac arrest in individuals with genetic heart disease or other cardiac abnormalities [31]. Some studies that examined the causes of sudden cardiac death in athletes emphasized that the effects of exercise on the heart may lead to an increased risk of sudden cardiac events [7,8,37]. 

Recreational sports activities have been reported as the activity with the highest incidence of sports-related sudden cardiac death [12]. In our retrospective study, most (81%) football-related sudden cardiac death cases involved recreational football, a similar finding to an autopsy-based study of football-related sudden cardiac deaths in Turkey between 2000 and 2005, which found 87% of cases aged 10–48 years occurred in recreational football [18]. A study that investigated sports-related sudden cardiac death reported that most cases occurred in non-elite competitions and recreational sports activities [10]. In addition, the lack of ambulances, medical personnel, and automated external defibrillators (AED) in Turkey for recreational football may be considered a factor increasing these deaths. Ozbilgin et al. [38] reported that while an ambulance is provided at professional team matches in Turkey, there were no ambulances at amateur matches and an AED was only available in 5.2% of these stadiums in Turkey. Moreover, it was reported that 90% of sudden cardiac death cases were associated with ventricular fibrillation. One of the most critical factors for survival after cardiac arrest is timely defibrillation [38]. Therefore, the use of an AED on recreational football fields where there are no medical personnel may help reduce fatalities. Future research should explore the feasibility and effectiveness of AEDs in preventing football-related sudden cardiac death in Turkey, including the feasibility and acceptability in Turkey of training first responders in cardiopulmonary resuscitation and correct AED use. 

### Strengths and Limitations

To the best of our knowledge, the present study is the most comprehensive epidemiological study on football-related sudden cardiac death in Turkey. Although autopsy-based studies have previously conducted in Turkey, they are limited to small sample sizes (*n* = 6 and *n* = 15) [17,18] as not all fatalities have confirmed causes of death by autopsy [17]. For this reason, data derived from media reports can be a viable alternative, as has been seen in other studies [10,19,20,21]. 

There are limitations associated with this research. Cases included in this study are sudden deaths deemed to be of cardiac origin from media reports only and may not be as accurate as case reports based on findings from an autopsy [21], or may be sudden deaths of unexplained causes. However, although media data may not capture all deaths, it should be noted that this method identifies more cases than autopsy-based or case studies. However, data on this issue in Turkey must be strengthened and strategies to improve national data collection, such as the formation of an out-of-hospital cardiac arrest registry may be worthwhile.

Mortality rates are calculated using population data and do not consider exposure to the activity. Participation data for football in Turkey by age group would allow for more accurate rates to be calculated, however this data is currently unavailable. The use of media reports as the data source limits the risk factor data that can be identified, such as the use of illicit substances and tobacco use. It should also be noted that risk factors for SCD may differ significantly between different populations, potentially impacting age-specific mortality rates presented in this study. Although the inclusion of deaths <1 h within playing football is a reasonable approach, it must be noted that media reports may be less likely to report football as an association if the death occurs before or after a sporting event. Additionally, we were not able to analyze clinical and ECG data of the decedents. 

## 5. Conclusions

Football-related sudden cardiac death results in 13 deaths on average each year in Turkey, most commonly impacting males aged 30 to 49 years participating in recreational football. Although the age-specific mortality rate for football-related sudden cardiac death is lower than all-cause mortality for males in Turkey, prevention of such deaths is still an important public health issue. Promotion among both the general community and general practitioners of the importance of cardiac health checks prior to participation in recreational football should be conducted. Similarly, future research should explore the feasibility and acceptability in Turkey of AEDs at football fields, combined with training of community members in their use. This study has also identified the need for detailed data on out-of-hospital cardiac arrest in Turkey.

## Figures and Tables

**Table 1:** Age-specific mortality rate, proportional mortality rate, and relative risk (95% confidence interval) of football-related sudden cardiac death in Turkey (2011–2019).

Age Group	Football-Related SCD *n* (%)	Age-Specific Mortality Rate ^a^	Proportional Mortality Rate ^b^	RR and 95% CI ^c^
10–19 years.	10 (8.5)	0.15	0.35	0.7 (0.3–1.7)
20–29 years.	23 (19.5)	0.36	0.57	1.6 (0.8–3.5)
30–39 years.	41 (34.7)	0.64	0.86	2.9 (1.4–6.1)
40–49 years.	35 (29.7)	0.67	0.37	3.1 (1.5–6.4)
50–59 years.	9 (7.6)	0.22	0.05	1
Total	118 (100)	0.41	0.30	-

SCD: sudden cardiac death, RR and 95% CI: relative risk and 95% confidence interval. ^a^ Age-specific mortality rate was calculated in units of deaths per 100,000 ratio of the number of football-related sudden cardiac death among those aged 10–59 years to the number of persons aged 10–59 years. ^b^ Proportional mortality was calculated units of deaths per 1000 ratio of the number of football-related sudden cardiac death among those aged 10–59 years to the number of deaths from all causes among those aged 10–59. ^c^ The age group 50–59 years was considered as the reference point for calculating RR and 95% CI.

**Table 2:** The phase of exercise and the level of football participation in which sudden cardiac deaths occur by age groups (2011–2019).

Age Group	Phase of Exercise						The Level of Football Participation			
	Before Exercise		During Exercise		After Exercise		Non-Elite Competitive		Recreational Football	
	*n*	%	*n*	%	*n*	%	*n*	%	*n*	%
10–19 years.	-	-	10	100	-	-	-	-	10	100
20–29 years.	1	4.3	16	69.6	6	26.1	2	8.7	21	91.3
30–39 years.	1	2.5	32	78.0	8	19.5	2	4.9	39	95.1
40–49 years.	-	-	32	91.4	3	8.6	1	2.9	34	97.1
50–59 years.	1	11.1	7	77.8	1	11.1	-	-	9	100
Total	3	2.5	97	82.2	18	15.3	5	4.2	113	95.8

Before exercise: warm-up phase, during the exercise: the stage in which the match is played, after exercise: the first hour after the end of the exercise. *n*: number.

**Table 3:** Percentage and incidence of football-related sudden cardiac deaths in Turkey by Seasons (2011–2019).

Season	Age Group					
	10–19 yr. *n* (%)	20–29 yr. *n* (%)	30–39 yr. *n* (%)	40–49 yr. *n* (%)	50–59 yr. *n* (%)	Total *n* (%)
Winter	3 (30.0)	6 (26.1)	7 (17.1)	7 (20.0)	3 (33.3)	26 (22.0)
Autumn	3 (30.0)	3 (13.0)	12 (29.3)	10 (28.6)	1 (11.1)	29 (24.6)
Summer	-	6 (26.1)	8 (19.5)	10 (28.6)	4 (44.4)	28 (23.7)
Spring	4 (40.0)	8 (34.8)	14 (34.1)	8 (22.9)	1 (11.1)	35 (29.7)

## Data Availability

Data may be made available upon reasonable request. Please contact data custodian Ali Işın (isin_ali@hotmail.com) for further information.
